# Preliminary Study on the Tears Oxidative Stress Status and Sleep Disturbances in Irritable Bowel Syndrome Patients

**DOI:** 10.1155/2020/4690713

**Published:** 2020-05-23

**Authors:** Ioana-Miruna Balmus, Roxana-Oana Cojocariu, Alin Ciobica, Stefan Strungaru, Roxana Strungaru-Jijie, Alina Cantemir, Catalina Galatanu, Lucian Gorgan

**Affiliations:** ^1^Department of Interdisciplinary Research in Science, Alexandru Ioan Cuza University of Iași, Carol I Avenue, No. 11, Iași, Romania; ^2^Department of Research, Faculty of Biology, Alexandru Ioan Cuza University of Iași, Carol I Avenue, 20A, Iași, Romania; ^3^Department of Biology, Faculty of Biology, Alexandru Ioan Cuza University of Iași, Carol I Avenue, 20A, Iași, Romania; ^4^Oftaprof Ophthalmological Clinic, Stejari Street, No. 54, Iași, Romania

## Abstract

According to the latest gastrointestinal disorders diagnostic criteria (ROME IV), the irritable bowel syndrome (IBS) is mainly characterized by the presence of abdominal pain and changes in intestinal transit. However, both sleep impairments and oxidative status changes (in patients' sera, mucosal level, and other body fluids) were reported IBS. Thus, in this study, we aimed to evaluate several aspects regarding the oxidative stress status in patients' tears as well as sleep disturbances by comparison with the intensity of IBS symptoms, as assessed by the visual analogue scale for irritable bowel syndrome (VAS-IBS). Ten IBS patients and fourteen healthy sex- and age-matched volunteers were recruited from the Oftaprof Ophthalmological Clinic (Iași, Romania). Visual analogue scale for irritable bowel syndrome and the Pittsburgh Sleep Quality Index (PSQI) questionnaires were administered to all the patients. Tear samples were collected using the Schirmer test procedure and were subjected to biochemical analysis—superoxide dismutase and glutathione peroxidase activities, malondialdehyde, and total soluble proteins levels were determined. Standard statistical analysis was applied. We found significant differences in oxidative stress marker dynamics in IBS patients as compared to healthy age- and sex-matched controls: increased superoxide dismutase activity (*p* = 0.02), increased malondialdehyde (*p* = 0.007), and total soluble proteins levels (*p* = 0.019). We found no significant differences in tear glutathione peroxidase activity in IBS patients as compared to healthy age- and sex-matched controls (*p* = 0.55). Furthermore, we observed that the oxidative stress tear markers are correlated with gastrointestinal symptoms severity (as evaluated by VAS-IBS) but not correlated to the sleep quality index and items (as evaluated by PSQI), with significant differences according to patient sex and IBS subtype stratification. In this way, this study brings additional evidence of the oxidative stress role in IBS pathology alongside the evaluation of tear fluid molecular dynamics in IBS for the first time in our best knowledge.

## 1. Introduction

It is now currently accepted that irritable bowel syndrome (IBS) is a chronic functional disorder which exhibits gastrointestinal and mood impairment symptoms [1]. According to the latest gastrointestinal disorders diagnostic criteria (ROME IV), IBS is mainly characterized by the presence of abdominal pain and changes in intestinal transit [2]. Also, the changes in mood and affective status could be associated with IBS, affective disorders being currently considered comorbidities in IBS [3].

In this way, it was shown that alongside the affective impairments, such as anxious and depressive moods, IBS patients could exhibit sleep impairments [4]. Thus, in a recent study of our group in which we discussed the incidence of sleep disorders and the mechanistical correlation with IBS, we concluded that sleep disturbances are rather a common symptom in IBS, whereas sleep disorders could be also considered comorbidities in IBS [5]. Furthermore, some studies reported significant differences in subjective sleep quality in IBS patients, as compared to healthy subjects [6–8].

Moreover, Waller et al. [9] argued the correlation between sleep disorders and certain ophthalmological diseases. In this way, they discussed the possible implications of sleep disorder and eye impairments including molecular changes occurring in glaucomic eye. Lee et al. [10] also demonstrated that the tear film could be impaired in sleep deprivation suggesting a correlation between sleep disorders and tear film consistency and functions. In this way, we recently showed that tear film impairments could be linked to oxidative stress in a certain ophthalmological disorder and that it could be modulated by surgical and antioxidant treatments [11].

Oxidative changes were also documented in several biological fluids in IBS patients [12–14]. In this way, oxidative stress was shown to be significant in both systemic and mucosal levels as a result of innate immune dysfunction in IBS pathogenesis [15]. Moreover, our group previously showed that oxidative stress could be an important component of IBS being present in the cerebral tissues in a significant correlation with the exhibited behaviour [16, 17].

Thus, in this study, considering the possible correlation between the sleep disturbances occurring in IBS and also the implication of oxidative stress in this pathology and the human eye's high sensibility to oxidative changes, it was our goal to evaluate several aspects regarding the oxidative stress status in IBS patients' tears. In this way, we also aimed to correlate the tears oxidative changes in the context of both IBS symptom severity (as assessed by the visual analogue scale for IBS) and sleep disturbances intensity (expressed as Pittsburgh Sleep Quality Index).

## 2. Patients and Methods

### 2.1. Patients and Groups

Ten IBS patients and fourteen healthy sex- and age-matched volunteers were recruited from the Oftaprof Ophthalmological Clinic (Iași, Romania). The mean age of the patients was 42.6 years, and the sex ratio was 50% females and 50% males (Table 1). All the recruited patients were divided into subgroups according to the IBS classification system, and the patients constituted 2 subgroups: IBS-D (diarrhoea predominant–IBS) and IBS-C (constipation predominant–IBS). Similarly, the mean age of the controls was 39.42 years, and the sex ratio was 50% females and 50% males (Table 1). No statistically significant differences were obtained in terms of age and sex ratio between the groups (*p*_age_ = 0.706).

Considering the specific needs of the study, several inclusion and exclusion criteria were formulated. The IBS patients' main inclusion criteria were the known functional gastrointestinal disorder diagnostic according to the ROME IV diagnostic criteria. Inclusion criteria for healthy control group comprised the absence of any known gastrointestinal, ophthalmological, neurological, psychiatric, chronic, or comorbid conditions and the sex and age matched to IBS group patients. Exclusion criteria for all the participants comprised history of any ophthalmological disorders, cardiovascular and cerebrovascular disease, hepatic and/or renal diseases, neurological or psychiatric conditions, malignancies and current supplementation by vitamins, polyunsaturated fatty acids and/or antioxidants, eye-rubbing habits, or smoking habits.

The study was conducted according to Declaration of Helsinki (1964), national and European regulations on Biomedical Research, and it was approved by the local committee. All the patients agreed and signed a written informed consent for their voluntary contribution in this study.

### 2.2. Visual Analogue Scale

Following Bengtsson et al.'s VAS-IBS scale [18, 19], we analysed the IBS symptoms intensity using the visual analogue scale for IBS (VAS-IBS). However, despite that Bengtsson et al. developed VAS-IBS according ROME II criteria [18, 19], we adapted and completed the VAS-IBS according to the ROME IV criteria and Romanian language [20].

### 2.3. Sleep Disturbances Evaluation

Similarly, for sleep disturbances evaluation, the Pittsburgh Sleep Quality Index (PSQI) scale available from http://www.goodmedicine.org.uk/ was applied to all the patients. PSQI assesses the sleep quality by differentiating “poor” from “good” sleepers. The scale is consisted in 7 items regarding subjective sleep quality, sleep latency, sleep duration, habitual sleep efficiency, sleep disturbances, use of sleep medication, and daytime dysfunction over the last month [21]. For each item, the scale provides answer variants (not during the past month=0, less than once a week=1, once or twice a week=2, and three or more times week=3) transposed to numerical scale from 0 to 3. Any missing answer should be counted as 0. The score calculation was carried out according to Buysse et al. [21]. The global sum of “5” or greater indicates a “poor” sleeper [7, 22, 23].

### 2.4. Sample Preparation

Tear samples were collected (during the morning hours) on Schirmer's strips according to the Schirmer test procedure without anaesthesia [24], following our previous experience [11]. The paper strips were placed on the lower lid pouch of each eye of the participant and allowed to collect tear samples for 5 minutes. Care was taken not to stimulate tear secretion during collection. Following the collection procedure, the Schirmer strips were weighted for tear volume quantification (30-50 *μ*l tears/eye), diluted with 500 *μ*l previously cooled PBS (phosphate-buffered saline), and stored in the Eppendorf tubes until analysis (-80°C).

### 2.5. Biochemical Analysis

Superoxide dismutase (SOD) and glutathione peroxidase (GPx) enzymatic activities, malondialdehyde (MDA), and total soluble proteins (PROT) concentrations were measured in tear samples in a UV-VIS spectrophotometric system (Analytik Jena Specord 210 Plus, Analytik Jena, Germany).

The total SOD enzymatic activity was determined using the spectrophotometric SOD Assay Kit (Sigma, Germany) according to the manufacturer's instructions. The assay kit measured the reaction inhibition rate (%) of water-soluble tetrazolium substrate and xanthine oxidase enzyme from 20 *μ*l biological fluid samples (tear/PBS dilutions). Following the incubation period (20 min at 37°C), the absorbance of the sample and blank reactions was read at 450 nm against distilled water. Enzymatic activity calculation was carried out using the mathematical formula suggested by the manufacturer's instructions.

Similarly, the GPx enzymatic activity was determined using the GPx Cellular Activity Assay Kit CGP-1 (Sigma, Germany) from 50 *μ*l biological fluid samples (tears/PBS dilutions). The enzymatic activity of GPx is in direct dependence with the amount of NADP+ obtained from the oxidation of NAPDH supplied in the reaction. Reaction dynamics were measured by reading of 340 nm light absorbance against reaction controls, according to the manufacturer's instructions.

Both SOD and GPx enzymatic activities were expressed as enzymatic units/mg proteins (specific activity); thus, the total soluble proteins concentrations were determined using the Bradford assay kit (Sigma, Germany) according to manufacturer instruction from 10 *μ*l biological fluid samples (tears/PBS dilutions).

Malondialdehyde concentrations were assessed using the Human MDA ELISA kit (CusaBio, P.R. China) based on the competitive inhibition enzyme immunoassay technique. The microplates are precoated with MDA-specific antibody, and a competitive inhibition reaction occurs between MDA and HRP-conjugated MDA with the precoated antibody specific for MDA. Following reaction stoppage, the microplates were read at 450 nm in a microplate reader system (Mindray MR-96A, Shenzhen, P.R. China). A total amount of 50 *μ*l of biological fluid samples (tears/PBS dilutions) was used to determine MDA concentrations which were expressed as *μ*g MDA/mg proteins.

### 2.6. Statistical Analysis

The biochemical analysis was performed twice for all the samples for statistical significance. Statistical analyses were carried out using the Minitab 19 software (Minitab Inc., 2019). Standard statistical analysis (One-way Single Factor ANOVA, student *t* test) was applied, and all results were expressed as mean ± SEM. Statistical correlations were also calculated using Pearson's correlation and Spearman's coefficient. *F* values for which *p* < 0.05 were regarded as statistically significant.

## 3. Results

### 3.1. Visual Analogue Scale–IBS

The scores of the visual analogue scale–IBS administered to the participants were significantly higher in the IBS patient group, as compared to age- and sex-matched healthy controls. The results of VAS-IBS on each of the items are presented in Table 1. In this way, the intensity of the physical symptoms such as abdominal pain (*p* < 0.001), diarrhoea (*p* = 0.021), constipation (*p* < 0.001), or bloating/gases (*p* < 0.001) was significantly increased in IBS patients.

**Figure 1:**
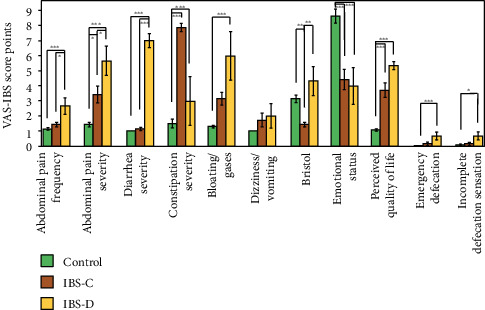
VAS-IBS item scores in IBS patients, stratified by IBS subtype. The results are expressed as the mean score of VAS scale (which ranges from 1 to 10, in direct correlation to severity) ± SEM (*n* = 14 for control, 10 for IBS, 7 for IBS-C, and 3 for IBS-D, ^∗^*p* < 0.05, ^∗∗^*p* < 0.01, ^∗∗∗^*p* < 0.001).

While analysing the results of the VAS-IBS items scores by stratification to IBS subtype, we observed that the abdominal pain frequency and intensity were significantly higher in IBS-D patients as compared to IBS-C patients (*p* < 0.05) and age- and sex-matched healthy controls (*p* < 0.001). Furthermore, the bloating and gases symptoms were significantly more intense in IBS-D patients (*p* < 0.001). No significant differences were observed regarding emotional status (item 4) and quality of life (item 5) between IBS subtypes (Figure 1) but compared to age- and sex-matched healthy controls (*p* < 0.001).

**Figure 2:**
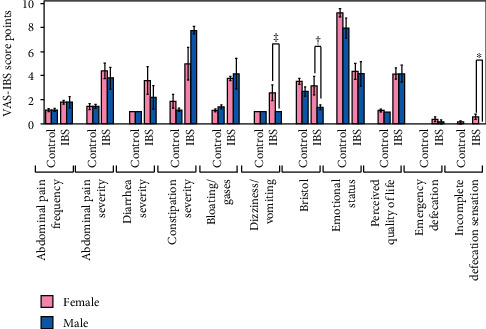
VAS-IBS item scores in IBS patients and age- and sex-matched healthy controls, stratified by sex. The results are expressed as the mean score of VAS scale (which ranges from 1 to 10, in direct correlation to severity) ± SEM (*n* = 14 for control, 10 for IBS, 7 for IBS-C, and 3 for IBS-D, ^∗^*p* < 0.05, ^†^*p* = 0.055, ^‡^*p* = 0.065).

While comparing the results of VAS-IBS items scores according to participants' sex and diagnostic, we observed no significant differences between VAS-IBS items scores in men and women of the control group, whereas the IBS women experienced more frequent nausea and vomiting (*p* = 0.065), diarrhoea (*p* = 0.055), and incomplete defecation sensation (*p* = 0.05) (Figure 2). No significant differences were observed regarding emotional status (item 4) and quality of life (item 5) between men and women.

### 3.2. Sleep Disturbances Evaluation

No significant differences were noted in PSQI regarding the age and sex of the participants. PSQI rating between 0 and 5 suggests normal sleep quality, whereas an index greater than 5 certainly characterizes a poor sleeper. In our study, we obtained a normal mean sleep quality index for the control group and increased PSQI for the IBS group which is significantly different from the first group (*p* < 0.001) (Table 1).

**Figure 3:**
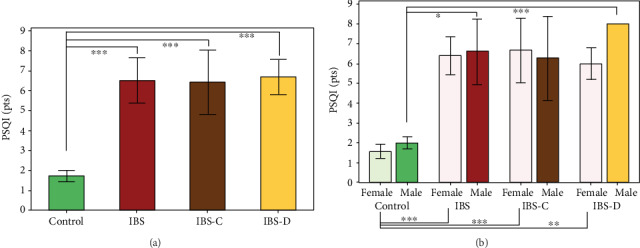
(a) Pittsburg Sleep Quality Index (PSQI) in IBS patients, as compared to healthy age- and sex-matched controls. The results are expressed as the mean score of PSQI ± SEM (*n* = 14 for control, 10 for IBS, ^∗∗∗^*p* < 0.001). (b) PSQI in IBS, stratified by patients' sex and IBS subtype. The results are expressed as means ± SEM (*n* = 14 for control, 10 for IBS, 7 for IBS-C, and 3 for IBS-D, ^∗^*p* < 0.05, ^∗∗^*p* < 0.01, ^∗∗∗^*p* < 0.001).

Regarding the IBS subtype stratification of the results, we found significantly increased PSQI in both IBS-C and IBS-D subgroups, as compared to control group (*p* < 0.001). However, no significant difference was observed between the PSQIs when comparing IBS-C with IBS-D (Figure 3(a)). The combined stratification of the groups regarding sex and IBS subtype revealed significant differences between males and female as a function to IBS subtype (Figure 3(b)).

Considering the analysis of the items regarding the bedtime habits (items 1-4), we observed that the IBS patients wake up considerately earlier, as compared to the control group (*p* = 0.022) and also spend more time trying to fall asleep (*p* = 0.026). While no significant differences were observed regarding the bedtime hour, the IBS patients declared that they sleep on average less than the age- and sex-matched controls (*p* = 0.02).

Afterwards, the participants were asked to evaluate the aspects they find inconveniently interfering with their sleep. Thus, problems such as prolonged falling asleep time, waking up in the middle of the night or early morning, the need to use the bathroom during the night, breathing uncomfortably, coughing or snoring loudly, feeling too cold/hot, having bad dreams, pain, or other reasons were evaluated and noted. The results showed that the most likely aspects to interfere with the participants sleep and impairing it were the prolonged falling asleep time (*p* = 0.012), waking up in the middle of the night or early morning (*p* < 0.001), coughing or snoring loudly (*p* = 0.032), and pain (*p* = 0.042). However, none of the participants acknowledged the use of prescribed or “over the counter” sleeping pills.

Regarding the last three items of PSQI, we also observed significantly different answers between the two studied groups. In this way, the IBS patients stated that they are making significant effort staying awake while driving, eating meals, or engaging in social activity (*p* < 0.01) and keeping up enough enthusiasm to get things done (*p* = 0.001). Furthermore, the IBS patients declared decreased overall rating to sleep quality, as compared to sex- and age-matched controls (*p* < 0.001).

### 3.3. Biochemical Analysis

During the biochemical analysis of the tear samples, no significant differences were obtained between the two eyes of the participants. And no significant differences were obtained between female and male participants' tear samples.

**Figure 4:**
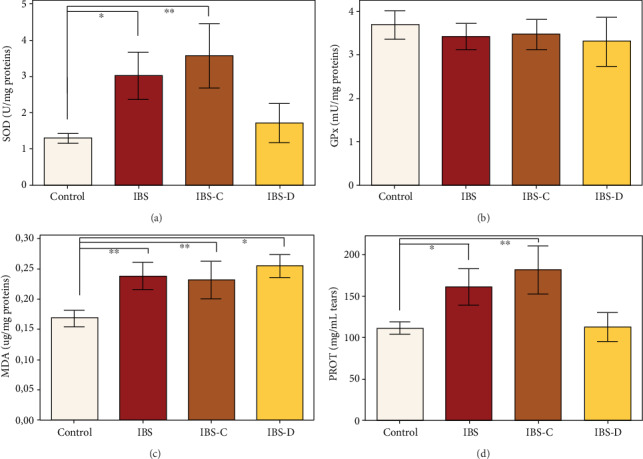
Studied oxidative stress markers in IBS patients (IBS=all patients; IBS-C and IBS-D=IBS group stratified by IBS subtype) and age- and sex-matched healthy controls, measured from tears. (a) Superoxide dismutase activity (U/mg proteins). (b) Glutathione peroxidase activity (U/mg proteins). (c) Malondialdehyde concentration (*μ*g MDA/mg proteins). (d) Total soluble proteins (mg/ml tears). The results are expressed as means ± SEM (*n* = 14 for control, 10 for IBS, 7 for IBS-C, and 3 for IBS-D, ^∗^*p* < 0.05, ^∗∗^*p* < 0.01).

Regarding the differences occurring between the two primary groups, we observed that in IBS patients' SOD activity (*F*(1, 46) = 5.84, *p* = 0.020), MDA levels (*F*(1, 46) = 7.85, *p* = 0.007), and total soluble proteins levels (*F*(1, 46) = 5.94, *p* = 0.019) significantly increased, as compared to sex- and age-matched healthy controls (Figure 4). No significant differences were observed for GPx activity by comparing the IBS patients group with the control group (*F*(1, 46) = 0.36, *p* = 0.55).

**Figure 5:**
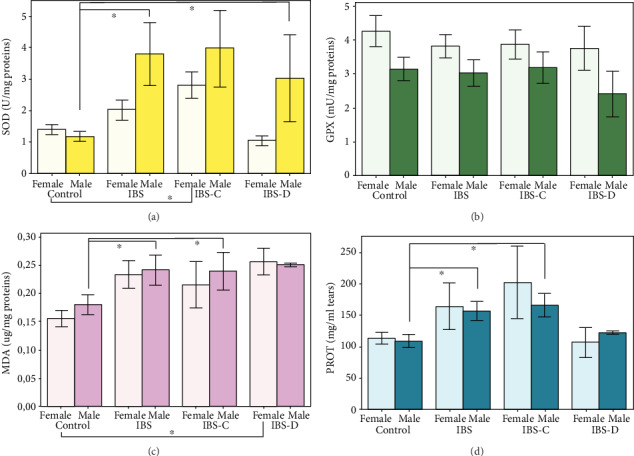
Studied oxidative stress markers in IBS patients' tears, stratified by patients' sex and IBS subtype. (a) Superoxide dismutase activity (U/mg proteins). (b) Glutathione peroxidase activity (U/mg proteins). (c) Malondialdehyde concentration (mmol MDA/mg proteins). (d) Total soluble proteins (mg/ml tears). The results are expressed as means ± SEM (*n* = 14 for control, 10 for IBS, 7 for IBS-C, and 3 for IBS-D, ^∗^*p* < 0.05).

Result analysis following stratification as a function to IBS subtype showed no statistical differences in oxidative stress marker levels between IBS-C and IBS-D patient samples. Result analysis following stratification as a function to participants' sex and group showed several differences which can be observed in Figure 5.

### 3.4. Correlation Analysis

Linear correlation analysis (Pearson's correlation) between oxidative stress markers in tears showed moderate negative correlations between MDA levels and GPx activity (*r* = −0.352, *p* = 0.014) and between MDA and total soluble proteins levels (*r* = −0.342, *p* = 0.017).

Also, significant linear correlations were found between tears' total SOD activity and constipation severity (*r* = 0.586, *p* = 0.003), Bristol's scale (*r* = −0.451, *p* = 0.027), self-assessment of quality of life (*r* = 0.440, *p* = 0.032), and painful events frequency (*r* = 0.444, *p* = 0.030). Tear GPx activity was correlated to the sensation of incomplete defecation (*r* = 0.462, *p* = 0.023), while MDA levels were correlated with self-assessment of quality of life (*r* = 0.492, *p* = 0.015), and painful events frequency (*r* = 0.505, *p* = 0.002). Total soluble protein levels were correlated with constipation severity (*r* = 0.430, *p* = 0.036) and with the frequency of events concerning the trouble staying awake while driving, eating meals, or engaging in social activity (*r* = 0.431, *p* = 0.035).

## 4. Discussion

The present knowledge on IBS pathophysiology admits several important pathological components, such as the gastrointestinal component, the neurological component, and the molecular component [25]. The molecular component is consisted in the oxidative and inflammatory changes which occur in and contribute to IBS symptomatology [26]. However, the IBS is characterized by only mild molecular changes [27], by contrast to inflammatory bowel disorder which is commonly known to exhibit a pregnant inflammatory component and important oxidative stress features which contribute to the tissular damages and disease relapse and remises [28]. This could be the reason why IBS remains a chronic yet functional gastrointestinal disorder which does not advance to life threatening stages and malignancies [28], and also, the less extensive molecular component of IBS could partially prevent the finding of molecular biomarkers which could lead to several difficulties in IBS diagnostic and research. In this context, this study showed that oxidative stress could be a permanent component of IBS pathology by being observable not only in patients' sera [14, 29], mucosal level [30], and possible metabolomic changes occurring in IBS patients urine [31] and faeces [32] but also in tear fluid.

Several studies suggested that oxidative stress plays an important role in IBS by being an active contributor to its development and its chronicity [12, 33]. Our recent studies in stress exposure-based IBS animal models showed that oxidative stress changes could also occur in the brain [16, 17] and intestinal tissues [34]. However, those observations could be discussed according to the studies which demonstrated that oxidative stress could be correlated to stress exposure, an important risk factor for IBS pathology [16, 35]. Thus, in this study, we showed that oxidative changes occur also in tear samples of IBS patients.

In this way, we observed that superoxide dismutase activity was upregulated in IBS patients in a significant manner. Moreover, we observed that this significant increase in activity could be relevant in male patients and in IBS-C subtype. This dynamic pattern in superoxide activity could suggest a prooxidant tendency since superoxide dismutase catalyses the transformation of one reactive oxygen species to another. Mete et al. [29] reported lower superoxide dismutase activity in IBS patients' sera, as compared to healthy controls. Similarly, we found decreased SOD activity in the brains of the IBS animal models we previously studied [16, 17]. However, it must be considered that the current determinations were made from a different body fluid, tear fluid, which is more susceptible to oxidative stress due to its role in eye protection and functions [11, 36, 37]. This could be the reason why tear superoxide dismutase activity and also malondialdehyde and total soluble proteins levels were found in our study to be dependent on the patients' sex and IBS subtype.

Moreover, we observed no significant differences in glutathione peroxidase activity in IBS patients' tears, as compared to healthy age- and sex-matched controls. In a recent study, Mendoza-Nunez et al. discussed the sex differences in oxidative stress markers, such as glutathione peroxidase and DNA damage, and concluded that the better antioxidant activity in women could be linked to the greater longevity [38]. In this way, our results also suggested that the antioxidant defence mechanisms are more efficient in women, due to the lack of significant changes in oxidative stress markers in IBS women and slightly positive dynamics, as compared to men. However, this could not explain why women are more prone to develop IBS and to exhibit more aggressive symptoms, as compared to men, as we observed during VAS-IBS evaluation.

Moreover, it is relevant to discuss the fact that previous studies acknowledge the role of sleep deprivation and impairment in ophthalmological disorders. In this way, our results showed no significant correlations between the oxidative stress markers and the Pittsburgh Sleep Quality Index score and items, except for the correlation between the troubled sleep due to pain versus SOD activity and MDA levels which is rather debatable since the addressed item on pain during sleep made no difference between general pain and gastrointestinal painful events (naturally occurring in IBS). By contrast, the oxidative stress markers were significantly correlated to VAS-IBS items scores, such as diarrhoea and constipation severity, Bristol's scale, and quality of life self-assessment. Thus, it seems that the oxidative stress changes in the tears of IBS patients could rather be a result of the gastrointestinal condition than a result of poor sleep quality or sleep deprivation.

The fact that we found significant sleep impairments in IBS patients as shown by the results of the PSQI questionnaire is yet additional evidence in the context of the cause-effect relationship of occurring impairments versus IBS pathology. It was previously discussed that sleep impairments and sleep disorders incidence in IBS could be frequent in the context of the IBS symptomatology burden on quality of life and mood [4, 39, 40]. In this context, our results showed that the poor quality of sleep could be due to the severity of the symptom, as shown by the significant correlations between the VAS-IBS items and PSQI items. In this way, we could propose that sleep impairments are in a causation-effect relationship with IBS symptomatology. However, several studies discussed the sleep impairments in IBS in the same contexts as the mood impairments which are now considered a stable component and comorbidities [41, 42]. Thus, it seems that oxidative stress, gastroenterological symptoms, and mood impairments are permanent IBS components exhibiting a complex relationship in a multifactorial manner.

## 5. Conclusion

This study provides additional evidence of oxidative stress implication in IBS pathology as we observed significant changes in superoxide dismutase activity, malondialdehyde, and total soluble proteins levels in the tear samples of the IBS patients, as compared to healthy age- and sex-matched controls. Yet, no significant differences were observed for the glutathione peroxidase activity. Furthermore, the results of this study suggested that the oxidative changes in the tear samples were not correlated to the sleep impairments occurring in IBS patients, except for the sleep impairment occurring due to painful events, but were significantly correlated to gastrointestinal symptomatology.

## Figures and Tables

**Table 1:** Demographic and pathophysiological description of the study groups.

	Study groups				*p* value^∗^
	Control (*n* = 14)	IBS (*n* = 10)			
ROME IV diagnostic criteria	—	Total	IBS–C	IBS–D	
		*n* = 10	*n* = 7	*n* = 3	
Age (means ± SEM, years)	39.43 ± 4.801^†^	42.60 ± 6.46^†^	37.57 ± 8.04	54.33 ± 8.68	^†^ *p* = 0.706
Sex ratio (F/M %)	50% M/ 50% F	50% M/50% F	57.15% M/42.85% F	33.3% M/66.6% F	
Pittsburg Sleep Quality Index (PSQI)	1.786 ± 0.28	6.5 ± 1.118^∗^	6.42 ± 1.601	6.66 ± 0.881	^∗^ *p* < 0.001
Visual analogue scale for IBS (VAS-IBS)					
Abdominal pain intensity	1.428 ± 0.17	4.1 ± 0.65^∗^	3.429 ± 0.68	5.67 ± 1.2	^∗^ *p* < 0.001
Diarrhoea	1^∗^	2.9 ± 0.91^∗^	1.143 ± 0.142	7 ± 0.57	^∗^ *p* = 0.021
Constipation	1.5 ± 0.35^∗^	6.4 ± 0.93^∗^	7.857 ± 0.34	3 ± 1.99	^∗^ *p* < 0.001
Bloating/gases	1.28 ± 0.12^∗^	4 ± 0.76^∗^	3.143 ± 0.50	6 ± 1.99	^∗^ *p* < 0.001
Nausea/vomiting	1^∗^	1.8 ± 0.46^∗^	1.714 ± 0.56	2 ± 0.99	^∗^ *p* = 0.053
Perception of psychological wellbeing (emotional status)	8.64 ± 0.57^∗^	4.3 ± 0.7^∗^	4.429 ± 0.84	4 ± 1.52	^∗^ *p* < 0.001
Daily life influenced GI problems (quality of life)	1.07 ± 0.07^∗^	4.20 ± 0.48^∗^	3.714 ± 0.60	5.333 ± 0.33	^∗^ *p* < 0.001

∗^†^Analysis of covariance.

## Data Availability

Data is available on request from the authors.
